# Fifth-Generation Cephalosporins Is Not Spared From Drug-Induced Liver Injury

**DOI:** 10.7759/cureus.50268

**Published:** 2023-12-10

**Authors:** Monish A Sheth

**Affiliations:** 1 Internal Medicine, Baylor College of Medicine, Temple, USA; 2 Internal Medicine, Baylor Scott & White Medical Center - Temple, Temple, USA

**Keywords:** herbal supplement adverse event, idiosyncratic hypersensitivity reaction, lft - liver function tests, rucam score, ceftaroline, cellulitis, cephalosporin induced, drug-induced liver injury (dili)

## Abstract

Ceftaroline fosamil is a fairly new parenteral cephalosporin antibacterial approved by the US Food and Drug Administration (FDA) for use in the treatment of acute bacterial skin and skin structure infections (ABSSSI). Drug-induced liver injury (DILI) is an important side effect profile to consider with antibiotic use. This case reports DILI in a previously healthy individual associated with a widely used antibiotic, ceftaroline, after only two doses. Discontinuation of the ceftaroline resulted in a resolution of DILI in a three-week period. Ceftaroline-induced liver injury is highlighted in this case report, along with a discussion of how we excluded other potential factors that can cause liver injury with the use of the Roussel Uclaf Causality Assessment Method (RUCAM) score. We also explained why other causes like hemochromatosis, Wilson's disease, celiac disease, and thyroid disease were unlikely.

## Introduction

Drug-induced liver injury (DILI) is an important cause of iatrogenic hepatic injury. Establishing a diagnosis of DILI requires careful exclusion of other etiologies and checking for the hepatotoxicity profile of suspected agents. A French population-based prospective study showed an annual estimated incidence of 13.9±2.4 DILI cases per 100,000 inhabitants. It has been extrapolated that nearly 44,000 patients in the United States will suffer from DILI each year [1]. Cefazolin has been most frequently linked to cholestatic jaundice; however, it is most commonly used cephalosporin as well. Hepatotoxicity is largely a class effect of the cephalosporins, even though it is idiosyncratic and rare. The pathophysiology of hepatic injury due to cephalosporins is not fully known, but similar to other penicillins, it is presumed secondary to hypersensitivity [2].

## Case presentation

**Figure 1:**
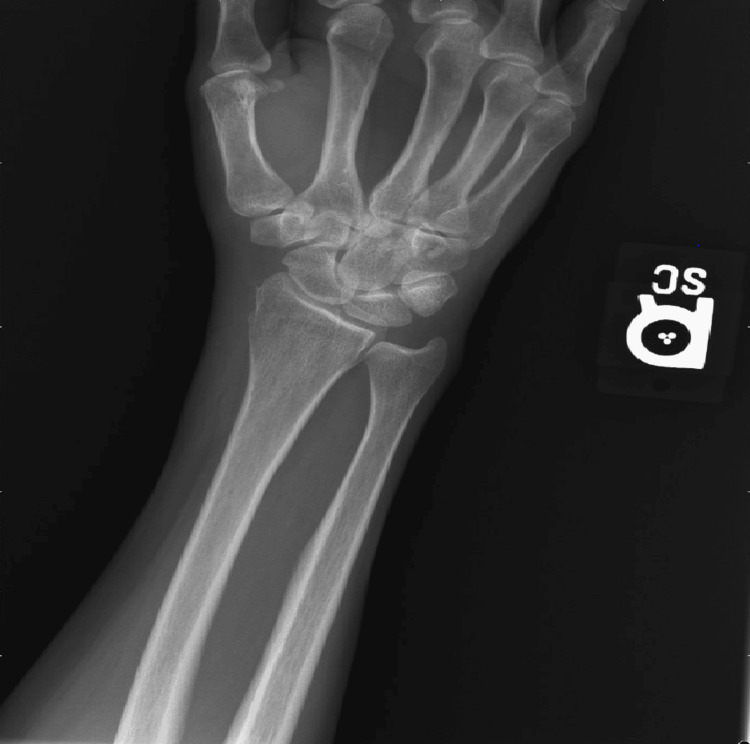
Right wrist X-ray Alignment is anatomic without fracture or lytic lesion. Joint spaces are well preserved. Soft tissues are normal. There is no cortical destruction or periosteal reaction to suggest plain film evidence of osteomyelitis.

A 52-year-old female presented with four days of right wrist pain, swelling, warmth, and redness. Vital signs were normal except for blood pressure of 150/83 mmHg. Physical exam was notable for erythema about the radial aspect of the wrist that extends from the base of the thumb across the wrist and into the distal forearm; mild edema about the wrist; no effusion, open wounds, drainage, bruising, laceration; tender to palpation most exquisitely over the radial aspect of the wrist, hand, and forearm; moderate warmth, no induration or fluctuance; compartments soft. Cardiac, abdominal, and pulmonary examinations were benign. On admission, the basic metabolic panel, complete blood count, and liver function test (LFT) were all normal. C-reactive protein was 12.7 mg/l (reference range: 0.0-3.2 mg/L). The right wrist X-ray was unremarkable, as shown in Figure 1. The patient was seen by orthopedic surgery and had arthrocentesis, which returned negative for infection or gout. The synovial fluid sample showed zero white blood cell count and no crystals. In the emergency department, she received antibiotics in the form of ceftaroline and pain control with intravenous morphine. Later, she did report some nausea, which was attributed to either antibiotic use or morphine use. LFT was checked after the patient received two doses of ceftaroline secondary to persistent nausea and mild abdominal discomfort, which resulted in aspartate transaminase (AST) of 674 IU/L, and alanine transaminase (ALT) of 730 IU/L, alkaline phosphatase of 209 IU/L and total bilirubin of 0.7 mg/dL. International normalized ratio (INR) was 1.0. Vital signs remained normal. The patient did not report taking high doses of acetaminophen or nonsteroidal anti-inflammatory drugs (NSAIDs). Ceftaroline was discontinued, and the antibiotic regimen was changed to vancomycin and piperacillin-tazobacam. Viral serologies, including hepatitis C antibody, hepatitis B surface antigen, and hepatitis A IgM antibody, were all negative. HIV was negative. She improved clinically with the decline in liver function tests over the next three days and resolved in the next 18 days, as shown in Table 1. She was discharged home on amoxicillin-clavulanate to finish her antibiotic course with primary care follow-up.

**Table 1:** LFT trends LFT - liver function test; SGOT - serum glutamic oxaloacetic transaminase; SGPT - serum glutamic pyruvic transaminase; ALT - alanine transaminase; AST - aspartate transaminase

LFT	One week prior to admission	Day of admission	Day one after the exposure	Day two after discontinuation	Day three after discontinuation	Day 18 after discontinuation
Bilirubin, total mg/dL; reference range: 0.2-1.2 mg/dL	0.3	0.4	0.7	0.4	0.3	0.3
Alkaline phosphatase [IU]/L; reference range: 34-130 [IU]/L	111	118	209	199	169	141
SGOT (AST) [IU]/L; reference range: 0-40 [IU]/L	14	14	674	217	96	15
SGPT (ALT) [IU]/L; reference range: 0-68 [IU]/L	9	7	730	438	293	14
Protein, total g/dL; reference range: 6.0-8.0 g/dL	6.8	6.8	5.9	6.2	5.8	7.5

With drastic improvement and normal liver function test on presentation, other causes like hemochromatosis, Wilson's disease, celiac disease, and thyroid disease (normal thyroid-stimulating hormone a week before presentation) were unlikely. The patient did not drink alcohol. Her Roussel Uclaf Causality Assessment Method (RUCAM) score in drug-induced liver injury was six (eight if we consider class effect with cephalosporin use) for ceftaroline and five for morphine. 

## Discussion

Diagnosis of DILI often necessitates having a high index of suspicion. Many drugs and over-the-counter medications/herbs/supplements have been linked with liver injury. DILI diagnosis can be easy if the patient has just started on one new medicine and has a temporal relation, but the real stipulation is that most times, it can be challenging to confirm, especially when the patient is taking multiple medications, and has medical comorbidities, illicit drug or alcohol abuse, or concomitant liver disease [3].

After reviewing PubMed and LiverTox, intravenous morphine was not considered the culprit, as opiates are unlikely to be associated with DILI in physiological doses to relieve pain. However, acute intoxication or very high doses have been linked to acute liver injury but potentially from shock-derived ischemic hepatitis [2].

Given the patient was presented with infection, septic shock-induced liver injury, which can be a common reason for elevated LFT, was not on differential given the patient was hemodynamically stable.

Alqahtani et al. collected data on DILI among 1019 patients between 2004 and 2012, and after undergoing careful causality assessment, 19 were attributed to cefazolin and 14 more to other cephalosporins (five first-generation, two second-generation, six third-generation, and one fourth-generation agent) [4]. Typically, the latent period for cephalosporin-induced DILI is one to three weeks with a self-limited moderate to severe clinical course [2]. However, it can happen as early as within 24 to 72 hours with a hypersensitivity-type reaction and if a re-exposure has occurred [2]. DILI has been reported after a single dose of cefazolin, with onset ranging from three to 23 days [4]. A case of DILI has been reported on the second day of being exposed to cefepime [5]. This is the first case to report DILI after one day of receiving ceftaroline.

## Conclusions

DILI is usually idiosyncratic and rare but needs vigilance in monitoring liver function tests to identify it early and stop the offending agent. It is important to rule out over-the-counter medications and supplements that patients might have started recently. Cephalosporins have a class effect, and the newer generation is not spared from these side effects. Even though hepatotoxicity usually occurs after taking several days of antibiotics, it can also happen after one dose, as described in the case. 
